# Association

**Published:** 1889-04

**Authors:** C. R. Butler

**Affiliations:** Cleveland, O.


### Association.

BY C. R BUTLER, M.D. D.D.S., CLEVELAND, O.

Read before the Mississippi Valley Dental Society, Cincinnati, March 7th, 1889.

I am prompted to present a few thoughts on the advantages of a community of effort in a given direction. To many this may seem a question so thoroughly settled that it has passed out of the category of dispute.

But from personal observation we find men that do not attend, and if we take them at their word, do not believe in dental associations, and yet they want to be called intelligent dentists. This is the age of associations, and all kinds are formed for mutual benefit in a broad sense.

The common excuse, that "I can not write an article nor speak," is no excuse why you should not be there as a listener, the babe has to learn to walk and talk; so with the most brilliant speaker and writer, he had to learn these things.

Some say, "Oh, there is no use to spend the time and money to attend these meetings, I can read at my leisure all that is worth having of the transactions as they are published in the journals."

We grant that the studious can gain much valuable information from the journals and text-books, but is it strictly honest with your fellows in the profession, to be getting and taking without giving anything in return? Your presence is worth something, and the use of the *one* talent is just as much of a duty as it is for him who has more.

And I want to say that many of the most valuable helps in practice are to be had only by attending these associations. Some persons have great skill in giving us pen pictures, but no doll can fill the place of the "meat baby."

The whole catalogue of modes in practice is made up of small things, suggestions of great value are often made that are so simple and familiar to the one making them that they are never formulated for the printer, consequently you must *see* or hear if you ever get them.

If all had staid at home, none could point with pride to the Mississippi Valley Association of Dental Surgeons, that has had an active existence of forty-five years.

Having attended yearly some association meeting, I ought to know the help that has come to me the past twenty-five years.

A good dinner is made more relishable by having some congenial companions at the table, than sitting down to enjoy it alone.

Many hints might be given by the writer to show the value of such meetings. We become acquainted and learn to love the noble men with whom we are associated in the effort to ameliorate our own and the condition of humanity at large.

The commonality believe in associations, and they read the daily papers, so they are liable to know that a meeting was had at a certain time. They may ask, were you there, and what new things did you see and hear? It would not sound very well to say, I never go; I don't believe in these associations. For even school girls might question you on physiology or some other matters pertaining to dentistry in such a manner that your ignorance of the subject would shake their faith in you as a safe dentist, though you may have been in practice for years. Some are careful students, while some are egotistical enough to say, "I know all the little modes and how's, and I don't believe in going to the societies or having any intercourse with other dentists."

But, my dear sir, you may be as brilliant as an electric lamp, and yet cast a shadow that the smallest weakling in the profession with his tallow dip, might light up.

